# Novel Anti-inflammatory Effects of Canagliflozin Involving Hexokinase II in Lipopolysaccharide-Stimulated Human Coronary Artery Endothelial Cells

**DOI:** 10.1007/s10557-020-07083-w

**Published:** 2020-10-13

**Authors:** Laween Uthman, Marius Kuschma, Gregor Römer, Marleen Boomsma, Jens Kessler, Jeroen Hermanides, Markus W. Hollmann, Benedikt Preckel, Coert J. Zuurbier, Nina C. Weber

**Affiliations:** 1 Department of Anaesthesiology, Laboratory of Experimental Intensive Care and Anesthesiology (L.E.I.C.A.), Amsterdam Cardiovascular Sciences, Amsterdam UMC, University of Amsterdam, Meibergdreef 9, Amsterdam, 1105 AZ the Netherlands; 2 Department of Anesthesiology, University Hospital Heidelberg, University of Heidelberg, Heidelberg, Germany

**Keywords:** Canagliflozin, Hexokinase 2, Endothelial cells, Inflammation

## Abstract

**Purpose:**

Vascular inflammation and disturbed metabolism are observed in heart failure and type 2 diabetes mellitus. Glycolytic enzyme hexokinase II (HKII) is upregulated by inflammation. We hypothesized that SGLT2 inhibitors Canagliflozin (Cana), Empagliflozin (Empa) or Dapagliflozin (Dapa) reduces inflammation via HKII in endothelial cells, and that HKII-dependent inflammation is determined by ERK1/2, NF-κB. and/or AMPK activity in lipopolysaccharide (LPS)-stimulated human coronary artery endothelial cells (HCAECs).

**Methods:**

HCAECs were pre-incubated with 3 μM or 10 μM Cana, 1 μM, 3 μM or 10 μM Empa or 0.5 μM, 3 μM or 10 μM Dapa (16 h) and subjected to 3 h LPS (1 μg/mL). HKII was silenced via siRNA transfection. Interleukin-6 (IL-6) release was measured by ELISA. Protein levels of HK I and II, ERK1/2, AMPK and NF-κB were detected using infra-red western blot.

**Results:**

LPS increased IL-6 release and ERK1/2 phosphorylation; Cana prevented these pro-inflammatory responses (IL-6: pg/ml, control 46 ± 2, LPS 280 ± 154 p < 0.01 vs. control, LPS + Cana 96 ± 40, p < 0.05 vs. LPS). Cana reduced HKII expression (HKII/GAPDH, control 0.91 ± 0.16, Cana 0.71 ± 0.13 p < 0.05 vs. control, LPS 1.02 ± 0.25, LPS + Cana 0.82 ± 0.24 p < 0.05 vs. LPS). Empa and Dapa were without effect on IL-6 release and HKII expression in the model used. Knockdown of HKII by 37% resulted caused partial loss of Cana-mediated IL-6 reduction (pg/ml, control 35 ± 5, LPS 188 ± 115 p < 0.05 vs. control, LPS + Cana 124 ± 75) and ERK1/2 activation by LPS. In LPS-stimulated HCAECs, Cana, but not Empa or Dapa, activated AMPK. AMPK activator A769662 reduced IL-6 release.

**Conclusion:**

Cana conveys anti-inflammatory actions in LPS-treated HCAECs through 1) reductions in HKII and ERK1/2 phosphorylation and 2) AMPK activation. These data suggest a novel anti-inflammatory mechanism of Cana through HKII.

**Electronic supplementary material:**

The online version of this article (10.1007/s10557-020-07083-w) contains supplementary material, which is available to authorized users.

## Introduction

Chronic vascular inflammation is a common early signature in patients with type 2 diabetes mellitus (T2DM) and is strongly associated with an increased risk of cardiovascular disease [1–3]. The metabolic profile of T2DM consists of hyperglycemia, which instigates a shift toward disturbed glucose utilization leading to accumulation of glycolytic intermediates and reactive oxygen species in endothelial cells [4]. Hexokinase (HKII) is an early glycolytic enzyme that facilitates glucose conversion to glucose-6-phosphate (G6P) leading to increased metabolic flux through glycolysis. However, increased expression and activity of HKII and upregulated glycolysis are observed in inflammatory conditions [5–8]. Blocking HKII ameliorates inflammatory signaling in activated isolated human cells and in small animal models of inflammation [5, 6, 8–11]. Targeting enhanced glycolysis via HKII inhibition may hence alleviate the inflammation that is involved in the vascular pathology of T2DM.

The kidney-targeted sodium-glucose cotransporter 2 (SGLT2) inhibitors Canagliflozin (Cana), Empagliflozin (Empa), and Dapagliflozin (Dapa) exhibited pronounced beneficial effects on cardiovascular outcome in patients with and without T2DM, including reductions in heart failure events, hospital admissions, and chronic kidney disease [12–15]. Recent data suggest that direct, SGLT2 unrelated, cardiovascular actions of SGLT2 inhibitors account, at least in part, for the reported cardiovascular benefits [16–21]. Interestingly, several of these studies suggest an anti-inflammatory mechanism underlying the positive clinical outcomes of SGLT2 inhibitors [18, 22, 23]. Others report that glucose uptake is hindered by SGLT2 inhibitors in a variety of cell types, including endothelial cells [24–26].

At the site of the endothelium, inflammation is best characterized by the release of pro-inflammatory cytokines and enhanced expression of adhesion molecules. Both facilitate leukocyte recruitment and translocation through the vascular wall leading to endothelial dysfunction [27]. The presence of leukocytes also promotes fibrosis and enables atherosclerotic plaque formation in the coronary circulation. In cell and animal models of diabetes as well as in patients with T2DM, elevated levels of circulating and endothelial inflammatory markers, such as interleukin 6 (IL-6) and vascular cell adhesion molecule 1, as well as markers of endothelial dysfunction are generally observed [28–32].

Diabetes mellitus associates with changes in the gut microbiome, increasing the leakiness of LPS through the gut wall, thereby further inducing tissue inflammation [33]. LPS can bind to the toll-like receptor 4 (TLR4) that is expressed on endothelial cells. TLR4 binding simultaneously activates nuclear factor-κB (NF-κB) and the mitogen-activated protein kinases (MAPK), including extracellular regulated kinases 1 and 2 (ERK1/2) [34]. In macrophages, activation of TLR4 by LPS shifts the cell metabolism to a glycolytic phenotype, with elevated glucose consumption, lactate production, and increased HKII expression and activity [7]. Furthermore, a central regulator of cellular metabolic pathways and an anti-inflammatory signaling protein is the adenosine monophosphate (AMP)-activated kinase (AMPK) [35, 36]. Previous studies have reported that SGLT2 inhibitors can activate AMPK, and that AMPK activation may contribute to the anti-inflammatory mechanism of these drugs [18, 19, 37]. Collectively, these findings suggest a strong association between cell metabolism and inflammation. Targeting metabolic intermediates of glycolysis to attenuate inflammation may be a key strategy to reduce early vascular abnormalities occurring in diabetes-associated cardiovascular disorders.

An anti-inflammatory action of SGLT2 inhibitors has not been previously linked to changes in endothelial glycolytic enzymes, such as HKII. Therefore, in this present study, we hypothesized that (1) SGLT2 inhibitors Cana, Empa, and Dapa reduce LPS-stimulated inflammation in human cardiac endothelial cells; (2) HKII is involved in the possible anti-inflammatory effects of the SGLT2 inhibitors; and (3) ERK1/2, NF-κB, and AMPK activity have a role in the HKII-dependent anti-inflammatory actions of SGLT2 inhibitors.

## Methods

### Cell Culture and Experimental Procedure

HCAECs were purchased from Promocell (Heidelberg, Germany) and grown in vascular basal cell medium with supplements (ATCC, Manassas, VA, USA), containing 5-ng/ml vascular endothelial growth factor, 5-ng/ml epidermal growth factor, 5-ng/ml fibroblastic growth factor, 15-ng/ml insulin-like growth factor 1, 10-mM L-glutamine, 0.75-U/ml heparin sulfate, 1-μg/ml hydrocortisone, 50-μg/ml ascorbic acid, 1% amphotericin B, 1% penicillin-streptomycin, and 10% FBS. Cells were grown at 37 °C in a Heracell™ 150i CO_2_ incubator (Thermo Fisher Scientific, Waltham, MA, USA). All experiments were performed with cells from passage 5 to 8 when they reached 80–90% confluency. At the start of each experiment, cells were pre-incubated overnight (16 h) with vehicle or different compounds, including SGLT2 inhibitors at concentrations 3- and 10-μM Cana, 1-, 3- and 10-μM Empa, 0.5-, 3- and 10-μM Dapa (all three from MedChem, Sollentuna, Sweden), 1-μM TAK-242 to inhibit LPS-induced TLR4 signaling, 50-μM PD-98059 to inhibit ERK phosphorylation (Cambridge Bioscience, Cambridge, UK), 100-μM A7869662 to activate AMPK, or a combination of two compounds, in serum-reduced (2% FBS) media. The next day, HCAECs were subjected to 1-μg/mL LPS (Sigma Aldrich, Saint Louis, MO, USA) with vehicle or intervention for 3 h.

### Transfection with Small Interfering RNA (siRNA) for HKII

Knockdown of HKII in HCAECs was performed as previously described [38, 39]. In short, cells in 6-wells plates with confluency between 50 and 80% were transfected with 20-nM siRNA for HKII (Art# 4390824, ID# S6560, lot# ASO2DUJU, Thermo Fisher Scientifics) or negative control (AM4611, Ambion by Thermo Fischer Scientifics) for 24 h using Lipofectamine RNAiMax (Invitrogen by Thermo Fischer Scientifics, Waltham, MA, USA) and in antibiotic- and antimycotic-free medium. Cells were passaged 24 h after the start of transfection at a ratio of 1:2. Thereafter, the cells were cultured in antibiotic- and antimycotic-free medium for 48 h before the media was collected for IL-6 levels determination and cells were lysed for western blotting. At end experiment, a confluency of 80–90% was reached by the cells.

### Western Blot

Whole cell lysates were collected directly at the end of the experiment. Briefly, cells were rinsed with ice-cold PBS and collected in lysis buffer, made from RIPA buffer (150-mM NaCl, 50-mM TrisHCl, 1% Nonidet P40, 0.25% sodium deoxycholate, and 0.1% SDS), supplemented with 1-mM phenylmethylsulfonyl fluoride, 2-mM Na_3_VO_4_, 1-mM dithiothreitol, 1-mM sodium pyrophosphate, 50-μM sodium fluoride, and a protease inhibitor mixture (17-μM leupeptin, 1-μM aprotinin, and 12-μM pepstatin (all three from Sigma Aldrich)). After centrifugation at 14000 g, 4 °C for 10 min, the supernatant was collected and stored at − 80 °C until use. Samples were sonicated on ice in repeated short cycles (5 s, energy mode, 20 Joules, 70% amplitude, repeated for 4 times) using the Low Power Ultrasonic Systems 2000 Lpt/LPe with microtip (Branson, Danbury, CT, USA).

Western blotting was performed as described previously [40]. Sample protein contents, determined with the Lowry method, were adjusted to the same concentration for each blot. After overnight incubation with primary antibodies against phospho-AMPK, AMPK, HKI, HKII, phospho-ERK, ERK, and GLUT1 (1:1000, all from CST, Danvers, MA, USA) and household protein GAPDH (CST, 1:5000), membranes were washed with phosphate-buffered saline (PBS) containing 0.1% tween (Sigma) and incubated with the complementary secondary antibody (IRdye, 1:5000, Li-Cor, Lincoln, NE, USA) for 1 h at room temperature before they were washed again. The membranes were scanned with the Odyssey CLx operator (Li-Cor) at auto-scan setting for dynamic range, 169-μm resolution and medium quality, and quantification of the bands was performed with Image Studio^TM^ Software (Version 5.2, Li-Cor). For quantification of the band signals, the signal from each band was normalized to the signal from the largest band on the membrane according to the manufacturer’s instructions, to reduce the chances of technical variation. Whole membrane scans used for the results sections are provided in the ESM (Electronic Supplementary Materials).

### Enzyme-Linked Immunosorbent Assay (ELISA)

The supernatant from each well was collected and spun at 250 g, 4 °C for 10 min. Levels of IL-6 were determined using ELISA (R&D Systems, Minneapolis, MN, USA) according to manufacturer’s instructions.

### Hexokinase Activity

HK activity was measured photospectrometrically in cell extracts at 25 °C with glucose-6-phosphate dehydrogenase, glucose, adenosine triphosphate, and nicotinamide adenine dinucleotide (NAD^+^), in the presence of rotenone to inhibit mitochondrial respiration [41]. The rate of NADH formation from NAD^+^ was determined over 180 s and used for the measure of total HK activity. HK activity was corrected for total protein concentration in each sample.

### Sample Size Calculation and Statistical Analyses

Four experiments were needed to detect a physiologically relevant difference of 25% between control and intervention, given a standard deviation of 10%, a power of 80%, and an *α* of 0.05. Data are presented as mean ± standard deviation (SD). The distribution of the data was tested using the Shapiro-Wilk test. Normal distributed data were tested by one-way ANOVA with Bonferroni post hoc testing or by a Student’s *t* test. Non-normally distributed data were tested with Kruskal-Wallis and Mann-Whitney U test with Bonferroni correction. In each experiment, we attempted to avoid experimental bias by single-well use by pooling two wells with cells. The number of experiments mentioned in the figures refers to the number of technical replicates with cells from two donors (PCS-100-020 from ATCC, LOT# 59885589 and C-12221 from PromoCell, LOT# 425Z0191.1). Graphs were created in Graphpad Prism 8 and statistical analysis was performed using IBM SPSS Statistics 25. The cut-off values for statistical significance were indicated in the figures by *, **, and *** for *p* < 0.05, *p* < 0.01, and *p* < 0.001, respectively.

## Results

### Cana, but not Empa or Dapa, Attenuates IL-6 Release in LPS-Induced HCAECs

**Fig. 1:**
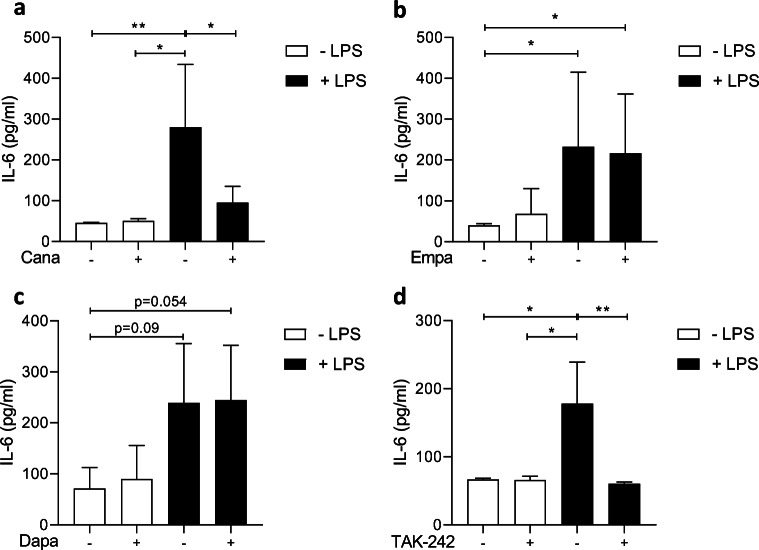
Cana, but not Empa or Dapa, attenuates IL-6 release in LPS-induced HCAECs. Cells were pre-incubated for 16 h with vehicle or an SGLT2 inhibitor and subsequently stimulated by 1-μg/mL LPS for 3 h with vehicle or an SGLT2 inhibitor. IL-6 release into the cell media was determined for Cana- (**a**, *n* = 4, 10 μM), Empa- (**b**, *n* = 6, 1 μM), and Dapa- (**c**, *n* = 6, 0.5 μM) treated HCAECs. TAK-242 (**d**, *n* = 3, 100 μM) served as a positive control to validate the inhibition of LPS-induced IL-6 release in our model. Data are presented as mean ± SD. *p < 0.05, **p < 0.01, tested by one-way ANOVA with Bonferroni correction

Exposing HCAECs to 1-μg/mL LPS for 3 h significantly increased IL-6 release. Cana (10 μM) almost completely blocked LPS-induced IL-6 release (Fig. 1a), whereas Empa (1 μM, Fig. 1b) or Dapa (0.5 μM, Fig. 1c) did not significantly alter IL-6 levels in the cell media. In addition, HCAECs treated with 10-μM Empa or Dapa exhibited no change in LPS-induced IL-6 release (ESM Fig. 1a–b). At a concentration of 3 μM, however, none of the SGLT2 inhibitors were able to lower LPS-induced IL-6 release (ESM Fig. 1c–e). No changes in IL-6 release were observed in healthy HCAECs exposed to an SGLT2 inhibitor (Fig. 1a–c). TAK-242 was able to nullify LPS-induced IL-6 release, confirming the involvement of TLR4 in IL-6 production by LPS (Fig. 1d).

### Cana Reduces IL-6 Release by Lowering HKII Expression

**Fig. 2:**
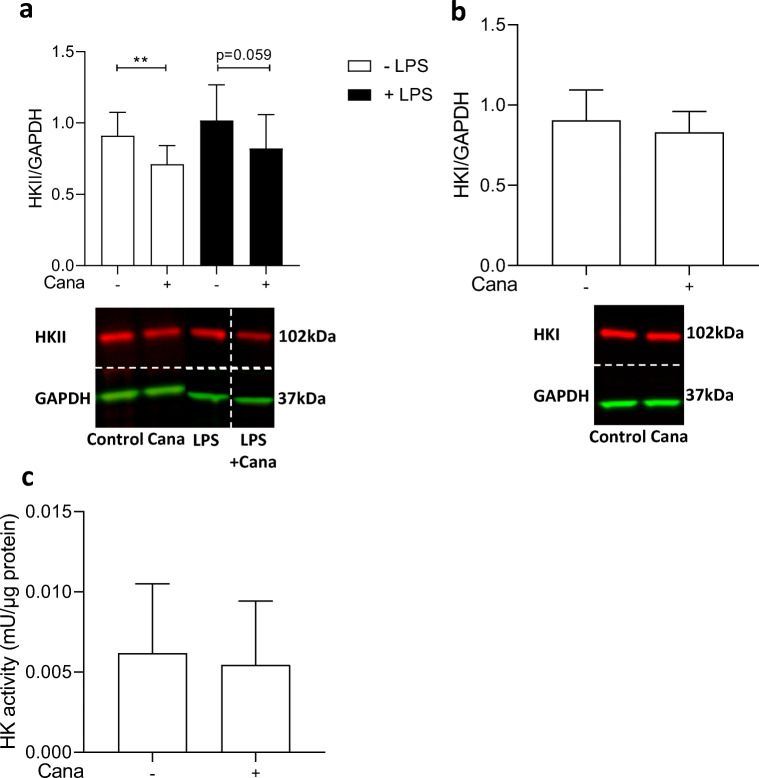
Cana reduces HKII, but not HKI, expression. Cells were exposed to vehicle or Cana (10 μM) for 16 h and subsequently subjected to 3 h LPS (**a**, *n* = 12). HKII and HKI levels (**b**, in non-stimulated, *n* = 7) were determined using infrared western blot. Representative bands are shown below each figure. Whole membrane scans are shown in the ESM. Total HK activity was detected photospectrometrically in whole cell lysate (**c**, *n* = 7–8). Data are presented as mean ± SD. *p < 0.05, tested by independent sample *t* test

Increased glycolysis, and in particular elevated HKII expression, is a signature of an activated inflammatory condition [5–11]. Incubating HCAECs for 19 h with 10-μM Cana resulted in a reduced expression of HKII in non-stimulated and LPS-stimulated HCAECs (Fig. 2a). In contrast, administration of 1-μM Empa or 0.5-μM Dapa did not affect HKII expression in non-stimulated HCAECs (ESM Fig. 2). Furthermore, the expression of HKI and the total HK activity were unaffected by Cana in non-stimulated HCAECs (Fig. 2b+c).

**Fig. 3:**
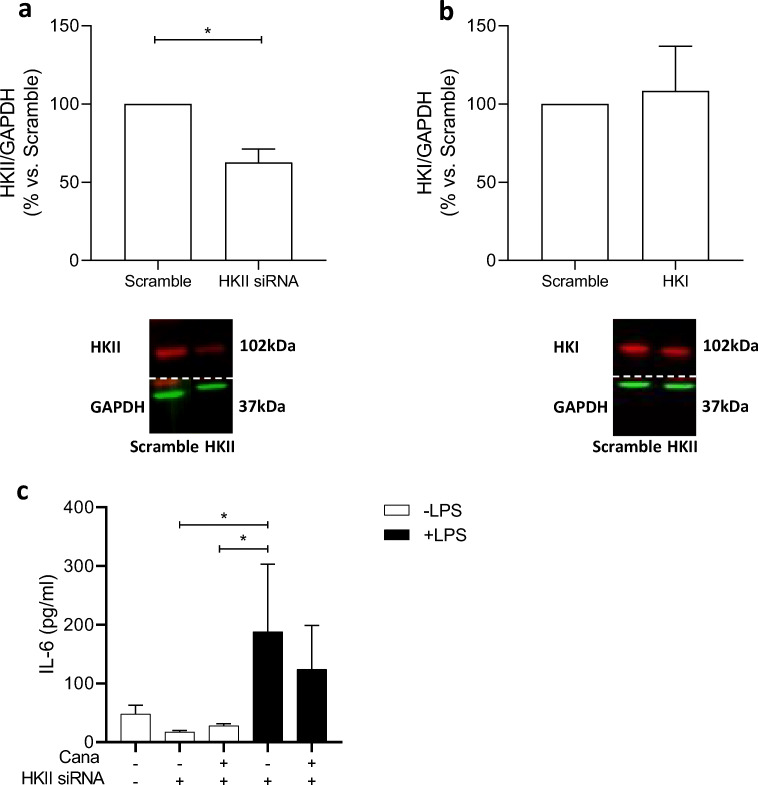
Knockdown of HKII caused partial loss of Cana-mediated IL-6 reduction HCAECs were transfected by siRNA for HKII or Scramble and pre-treated at *t* = 72 h post-transfection with vehicle or 10-μM Cana for 16 h and subsequently exposed to 0- or 1-μg/mL LPS with vehicle/Cana. Knockdown of HKII (**a**, *n* = 4) was achieved without change in HKI expression (**b**, *n* = 4). Representative bands are shown below each figure. Whole membrane scans are shown in the ESM. IL-6 release by Scramble vs. HKII knockdown cells in the presence of Cana/vehicle, with or without LPS (**c**). Data are presented as mean ± SD. *p < 0.05, tested by *t* test (**a**–**b**) and one-way ANOVA with Bonferroni correction (**c**)

Using siRNA transfection, knockdown of HKII by 37 ± 9% was achieved (Fig. 3a), without affecting HKI expression (Fig. 3b). Incubation with LPS caused augmented IL-6 release in siRNA HKII-treated HCAECs; however, this time Cana was unable to significantly reduce LPS-induced IL-6 release (Fig. 3c), indicating the involvement of HKII in the attenuating effect of Cana on cytokine release.

### Inhibition of LPS-Induced ERK Phosphorylation by Cana Is Mediated by HKII

**Fig. 4:**
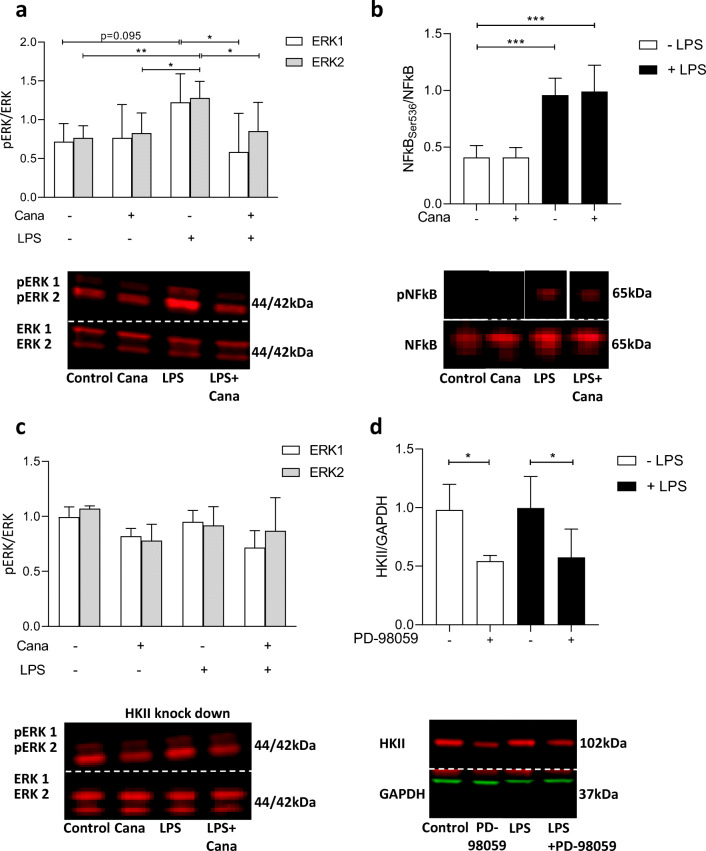
Cana inhibits LPS-induced ERK phosphorylation but does not affect NF-κB. ERK1/2 phosphorylation (**a**, *n* = 8) and NF-κB phosphorylation (**b**, *n* = 7) were measured in LPS stimulated and non-stimulated HCAECs. ERK1/2 phosphorylation was also determined in HKII knockdown HCAECs exposed to Cana and/or LPS (**c**, *n* = 4). HKII expression was measured in cells subjected to PD-98059 (**d**, *n* = 5). Representative bands are shown below each figure. Whole membrane scans are shown in the ESM. Data are presented as mean ± SD. *p < 0.05, **p < 0.01, ***p < 0.001, tested by one-way ANOVA with Bonferroni correction

To further explore downstream effects of Cana on LPS-induced IL-6 generation, we investigated whether Cana affected changes in the phosphorylation of ERK1/2 and NF-κB. First, LPS caused augmented ERK1/2 phosphorylation and Cana reduced LPS-induced ERK1/2 phosphorylation (Fig. 4a). While NF-κB phosphorylation was also increased with LPS, Cana did not affect NF-κB phosphorylation (Fig. 4b).

To examine the possible involvement of HKII in LPS-induced ERK1/2 phosphorylation, we investigated LPS effects on ERK1/2 in cells treated with siRNA for HKII. Reducing HKII completely mitigated LPS activation of ERK1/2 (Fig. 4c). As a consequence, Cana was without effect on LPS-induced ERK1/2 phosphorylation. These data indicate that decreasing HKII is upstream of ERK1/2 phosphorylation by Cana. Conversely, using PD-98059 to inhibit ERK1/2 phosphorylation in HCAECs, we also observed reduced HKII expression in HCAECs (Fig. 4d), indicating HKII reductions also occur downstream of ERK1/2 phosphorylation. These data suggest that reducing HKII expression blocks LPS-induced ERK1/2 phosphorylation, and that reduced ERK1/2 activity leads to lower HKII levels, irrespective of LPS treatment. ERK 1/2 phosphorylation and HKII reductions are intrinsically intertwined.

### Cana Induces AMPK Phosphorylation in Healthy and LPS-Stimulated HCAECs

**Fig. 5:**
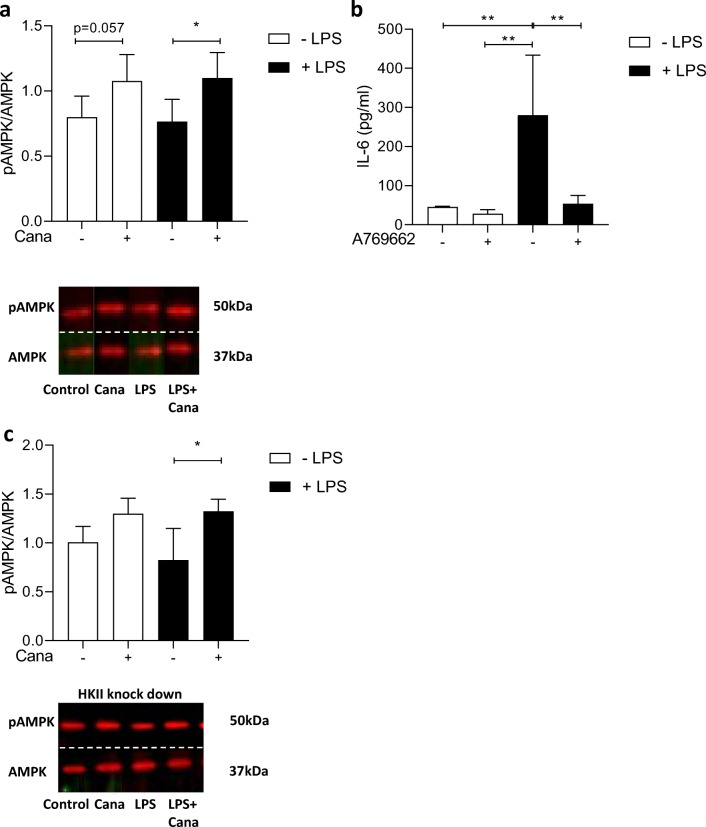
Cana induces AMPK phosphorylation, independent of HKII, in LPS-stimulated HCAECs. Cells were pre-incubated for 16 h with vehicle or Cana (10 μM) and subsequently stimulated by 1-μg/mL LPS for 3 h with vehicle or Cana. Phosphorylation of AMPK at site threonine 172 by Cana was determined in normal HCAECs (**a**, *n* = 7). Activation of AMPK by A769662 inhibits LPS-induced IL-6 release (**c**, *n* = 5). In HKII knockdown HCAECs, Cana still phosphorylated AMPK (**c**, *n* = 4). Representative bands are shown below each figure. Whole membrane scans are shown in the ESM. Data are presented as mean ± SD. *p < 0.05, tested by one-way ANOVA with Bonferroni correction

Previous studies have suggested the activation of AMPK as a contributing factor to SGLT2 inhibitor’s anti-inflammatory actions in various cell types [18, 19, 25], offering the possibility that Cana’s reducing effect on IL-6 production can also be mediated through AMPK activation. However, it is still unknown whether HKII is involved in AMPK effects on inflammation. Therefore, we investigated whether Cana activated AMPK, if AMPK activation resulted in reduction of IL-6 release, and if AMPK activation was dependent on HKII. First, we show that Cana phosphorylated AMPK at Thr172 in LPS-induced HCAECs (Fig. 5a). Second, activation of AMPK by A769662 (ESM Fig. 3a) leads to inhibition of LPS-induced IL-6 release (Fig. 5b). Furthermore, Cana administration resulted in increased AMPK phosphorylation in HKII knockdown HCAECs exposed to LPS (Fig. 5c), indicating that HKII is not involved in activation of AMPK by Cana. Empa and Dapa did not enhance AMPK phosphorylation in either non-stimulated or LPS-stimulated HCAECs (ESM Fig. 3b and c).

## Discussion

**Fig. 6:**
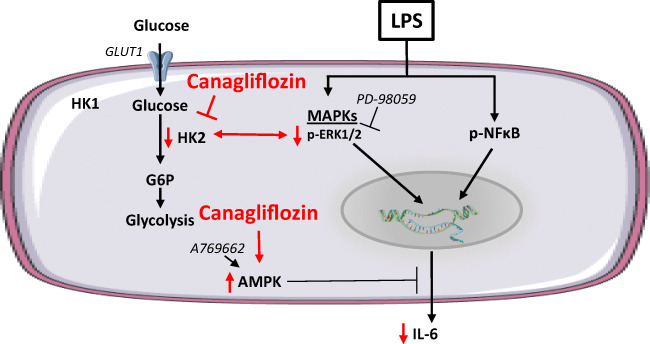
Summary of Cana effects in LPS-stimulated HCAECs. Cana reduces IL-6 release, at least in part, by lowering HKII and blocking ERK1/2 activation. Cana also activates AMPK, and AMPK activation is associated with reduced LPS-induced IL-6 release in HCAECs. Effects of Cana are indicated by red marks

The main findings of the present study are that the SGLT2 inhibitor Canagliflozin alleviates the release of the pro-inflammatory cytokine IL-6, lowers HKII expression, inhibits ERK1/2 phosphorylation, and activates AMPK in LPS-stimulated and/or non-stimulated HCAECs. The other two SGLT2 inhibitors Empa and Dapa did not show a significant reduction in IL-6 release, HKII expression, and AMPK activity in the model used. Reduced HKII expression seems to be associated with Cana’s anti-inflammatory effect by mediating LPS-induced IL-6 release and ERK1/2 phosphorylation, but not AMPK activation. These data suggest that under normoglycemic and inflammatory conditions, Cana exerts an anti-inflammatory activity by lowering HKII. A graphic summary of our findings is provided in Fig. 6.

### Anti-inflammatory Actions of Cana

We observed that the SGLT2 inhibitor Cana, but not Empa or Dapa, directly reduces the release of the pro-inflammatory cytokine IL-6 by LPS-activated endothelial cells. Our data correspond well with previous studies, showing that Cana reduces IL-6 release in IL-1β-stimulated human endothelial cells and in LPS-stimulated macrophages [18, 42]. However, the effect of Cana on IL-6 release reduction was absent at a lower concentration of 3 μM, suggesting that Cana may only be effective in attenuating IL-6 release at the higher concentrations. While we observed no direct cell effect of Empa or Dapa on LPS-induced IL-6 release, attenuated markers of endothelial dysfunction, i.e., ROS and nitric oxide, have been reported in activated endothelial cells exposed to Empa or Dapa, but without change in the expression of adhesion molecules [22, 23]. In the present study, we cannot rule out that Empa and Dapa do affect LPS-induced IL-6 release due to the higher variations observed in the experiments using Empa or Dapa. An explanation for the divergence between the effects of Cana vs. Empa or Dapa on LPS-induced cytokine release could be the different concentrations used. Cana was used at 10 μM, whereas Empa and Dapa were used at 1 μM and 0.5 μM, respectively, which are considered clinically relevant concentrations. However, using Empa or Dapa at 10 μM did also not cause reduced LPS-induced IL-6 release. Notably, the half maximal inhibitory concentration (IC50) of Cana for SGLT1 is estimated to be 710 nM, while Empa and Dapa have an IC50 for SGLT1 of 8300 nM and 1400 nM, respectively [43]. Thus in this context, SGLT1 and perhaps also other SGLT isoforms might be inhibited by Cana with the concentrations used in the present study.

One hypothesis for the direct cellular effects of SGLT2 inhibitors is the off-target inhibition of the Na^+^/H^+^ exchanger 1 (NHE1). NHE1 activity is enhanced in endothelial cells exposed to LPS [44]. Inhibition of NHE1 by a variety of different NHE inhibitors reduced LPS-induced apoptosis, cytokine production, and NF-κB activation [44, 45]. Cardiac NHE1 inhibition by SGLT2 inhibitors was reported as a class effect, showing similar inhibitory potentials for NHE1 by Cana, Empa, and Dapa [16]. Accordingly, NHE1 might have been activated by LPS stimulation, and Cana might have inhibited NHE1. Yet, since we observed different outcomes of Cana, Empa, and Dapa on LPS-induced cytokine release, NHE1 inhibition could not account for the anti-inflammatory effects of Cana in our model.

LPS induces inflammation after binding to TLR4 and activating downstream signaling events. These events lead to NF-κB activation and its translocation to the cell nucleus to stimulate production of pro-inflammatory molecules [27, 34, 45]. Concomitantly, MAPK that are involved in inflammatory processes, including ERK1 and ERK2, become activated [34]. In the present study, Cana inhibited LPS-induced ERK1/2 phosphorylation in HCAECs. Yet, since Cana does not affect NF-κB activation, which is observed in the present study as well as by other investigators [18], Cana may not act on the classical LPS-pathway upstream of both ERK1/2 and NF-κB. It is reported that Cana inhibits cell proliferation at clinically relevant concentrations, including the concentration used in the present study (10 μM) [18, 24, 46]. Cana’s anti-proliferative effect could be explained by a reduction in ERK1/2 activity, since ERK1/2 not only exerts pro-apoptotic and pro-inflammatory actions but also plays a role in proliferation and survival pathways [47]. In summary, reduction of ERK1/2 activity by Cana is observed during LPS stimulation, which may to some degree be related to the anti-inflammatory actions of Cana, although more research is needed to understand the role of ERK1/2 in the functional effects of Cana.

### Hexokinase as Target to Reduce Inflammation

Enhanced glycolysis is a central phenomenon in inflammation induced by LPS. A recent study showed that inflammation was associated with enhanced endothelial glycolysis and accumulation of glycolytic intermediates in human aortic endothelial cells [48]. HKII is one of several rate-limiting enzymes of glycolysis. HKII has been previously reported to be upregulated during inflammatory processes. In primary astrocytes exposed to hypoxia, HKII expression and activity were increased [49]. LPS strongly induced HKII gene expression after 24 h in human monocyte-dendritic cells [7]. In the present study, knockdown of HKII caused partial loss of Cana-mediated IL-6 reduction in HCAECs. Targeting hexokinase and therefore inhibiting glycolytic overload was previously proposed as a treatment strategy against inflammatory responses [8, 9]. We observed that Cana was able to reduce HKII expression in healthy HCAECs and LPS-stimulated HCAECs. We did not see an upregulation of HKII expression by LPS in our model, but this is likely due to the short time period that the HCAECs were exposed to LPS (3 h) as compared with previous studies showing enhanced HKII after 12 h or 24 h of LPS stimulation [6, 7]. To investigate whether reduction of HKII expression was associated with the anti-inflammatory effects of Cana, Cana effects were studied in partial HKII knockdown cells. While IL-6 release was still induced by LPS, Cana was unable to attenuate IL-6 release under reduced HKII conditions. This may imply that reduced LPS-induced IL-6 release by Cana is, at least partly, mediated by HKII. Furthermore, silencing HKII abrogated LPS-induced ERK1/2 phosphorylation, suggesting that HKII plays an important role in the induction of ERK1/2 activation during inflammation (or vice versa). Our data propose that Cana’s effect on reduced ERK1/2 phosphorylation and IL-6 release during inflammation is, at least partly, related to Cana’s effect on reduced HKII expression.

### AMPK Activation by Cana

Activation of AMPK by SGLT2 inhibitors have been previously reported [19, 25, 42]. We observed that Cana activated AMPK phosphorylation in LPS-stimulated HCAECs. Furthermore, AMPK activation by A769669 resulted in reduced LPS-induced IL-6 release. Cana was shown to reduce inflammation in IL-1β stimulated human endothelial cells [18], an effect that was at least partially associated with AMPK activation. The same authors reported that activation of AMPK by Cana was associated with inhibition of mitochondrial complex 1 inhibition in human kidney cells, mouse hepatocytes, and fibroblasts [25]. In LPS-stimulated immune cells, Cana at concentrations between 10 and 40 μM exhibited anti-inflammatory effects, reduced glucose metabolism, and promotes AMPK activation [42]. Cana’s inhibition of glucose metabolism is in line with our observation that HKII is reduced by Cana. Cana was still able to induce AMPK phosphorylation in HKII knockdown HCAECs stimulated with LPS, suggesting that Cana’s effect on AMPK is not mediated by HKII. HK expression and AMPK activity have been reported to be negatively related with each other in inflammatory conditions and may both be effective targets to alleviate inflammation-induced endothelial dysfunction [8, 9, 35]. Our data show that Cana activates AMPK and that AMPK activation reduces LPS-induced cytokine release. Furthermore, reduced HKII expression by Cana is not responsible for the increased AMPK phosphorylation; thus, it is likely that Cana increased AMPK activity through a different pathway or that Cana activates AMPK prior to Cana’s HKII lowering effect.

Empa and Dapa have primarily been related to direct AMPK activation in cardiac cells [19, 37, 50]. In our experiments, only Cana, not Empa and Dapa, induced AMPK phosphorylation in human cardiac endothelial cells (supplementary data), which is also reported in another endothelial cell study [18]. Activation of AMPK by SGLT2 inhibitors may possibly depend on the cell type and species origin (human cardiac endothelial cells vs. mouse cardiac fibroblasts and cardiomyocytes).

Finally, Cana has been found to activate autophagy in non-endothelial cell types [42]. The effects of Cana on autophagy in endothelial cells have not been studied. Our observations that Cana inhibits ERK1/2 and activates AMPK suggest that Cana increases autophagy in endothelial cells.

## Limitations and Conclusion

This study was only performed in non-diabetic HCAECs to understand the anti-inflammatory actions of SGLT2 inhibitors. We performed experiments in HCAECs that originate from two donors; therefore, the generalization of the results might be problematic and more experiments from different cell batches, types, and donors are needed to further validate our results. Nonetheless, the use of a single donor in human cell studies seems to be commonplace (e.g., in Hela cells [51], A549 cells [52], and HCAECs [53–56]). The effects of LPS on cellular stress were only investigated at a single time point in the present study. In our experiments, we assessed HKII expression in the cell lysate. The cellular location of HKII is crucial for its detrimental or protective function and its effects on cell metabolism [57]. Future studies should investigate the effect of Cana on the cellular localization of HKII, i.e., whether Cana affects mitochondrial bound HKII as well as cytosolic HKII.

In conclusion, Canagliflozin’s direct anti-inflammatory actions in human cardiac endothelial cells are associated with reduced hexokinase 2 expression.

## Electronic supplementary material

ESM 1 (PPTX 7375 kb)
