# Supplementary material for: Novel Anti-inflammatory Effects of Canagliflozin Involving Hexokinase II in Lipopolysaccharide-Stimulated Human Coronary Artery Endothelial Cells
Source: Cardiovasc Drugs Ther. 2020 Oct 13;35(6):1083–94. doi: 10.1007/s10557-020-07083-w (PMC8578058; doi:10.1007/s10557-020-07083-w)
Supplement: Supplementary file 1 — (PPTX 7375 kb) [file 10557_2020_7083_MOESM1_ESM.pptx]

## Slide 1
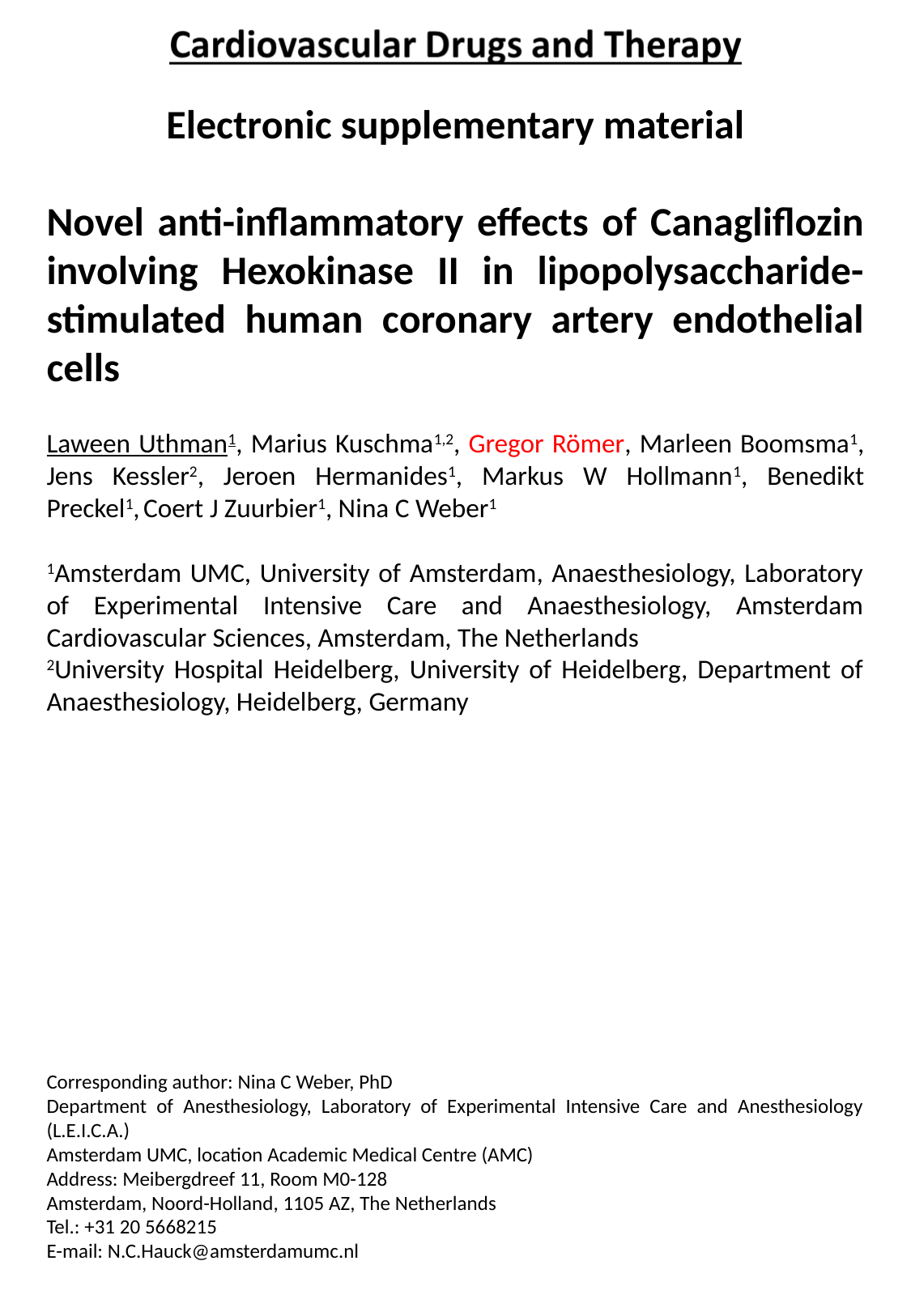

Electronic supplementary material
Novel anti-inflammatory effects of Canagliflozin involving Hexokinase II in lipopolysaccharide-stimulated human coronary artery endothelial cells
Laween Uthman1, Marius Kuschma1,2, Gregor Römer, Marleen Boomsma1, Jens Kessler2, Jeroen Hermanides1, Markus W Hollmann1, Benedikt Preckel1, Coert J Zuurbier1, Nina C Weber1
1Amsterdam UMC, University of Amsterdam, Anaesthesiology, Laboratory of Experimental Intensive Care and Anaesthesiology, Amsterdam Cardiovascular Sciences, Amsterdam, The Netherlands
2University Hospital Heidelberg, University of Heidelberg, Department of Anaesthesiology, Heidelberg, Germany
Corresponding author: Nina C Weber, PhD
Department of Anesthesiology, Laboratory of Experimental Intensive Care and Anesthesiology (L.E.I.C.A.)
Amsterdam UMC, location Academic Medical Centre (AMC)
Address: Meibergdreef 11, Room M0-128
Amsterdam, Noord-Holland, 1105 AZ, The Netherlands
Tel.: +31 20 5668215
E-mail: N.C.Hauck@amsterdamumc.nl

## Slide 2
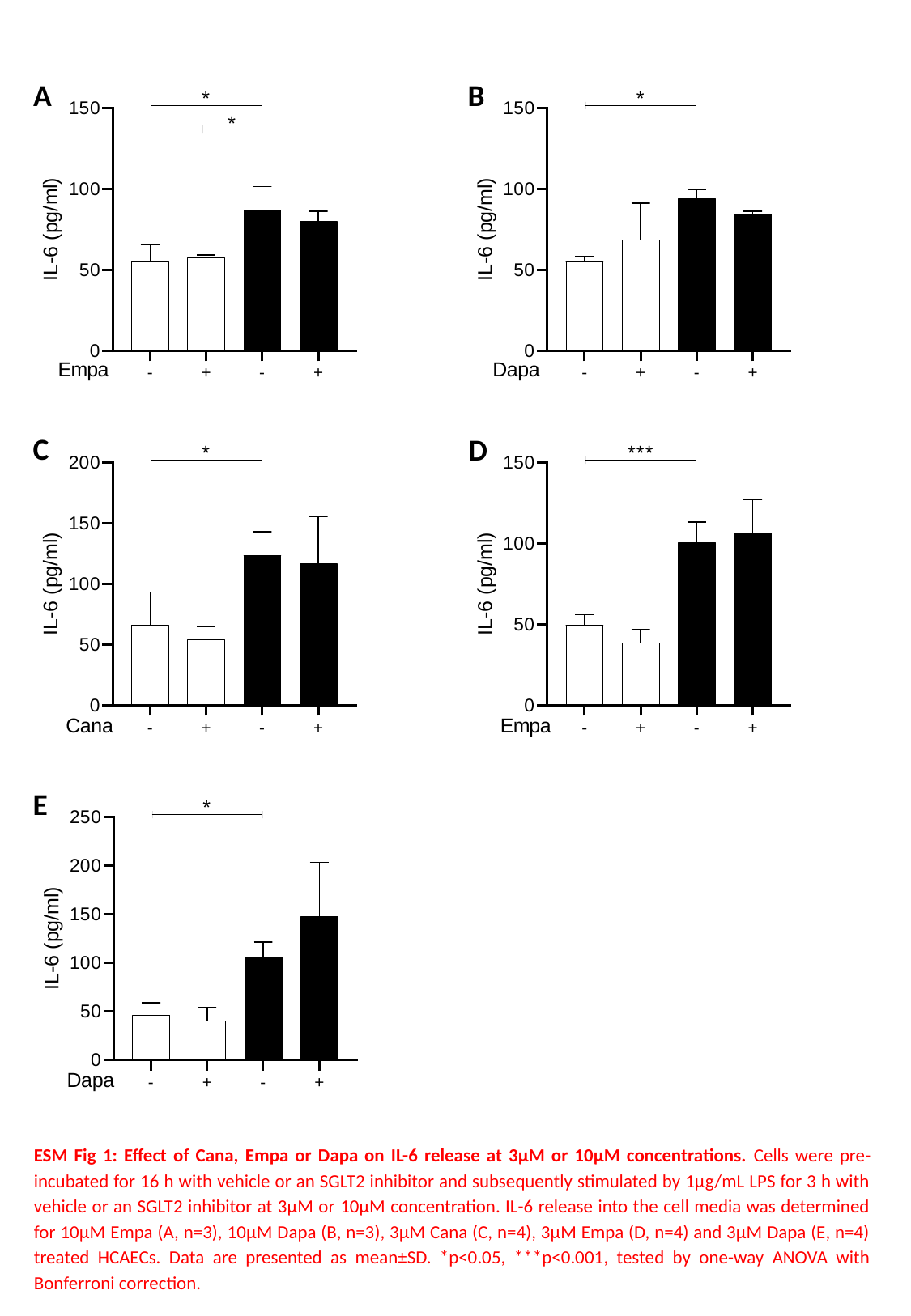

A
B
C
D
E
ESM Fig 1: Effect of Cana, Empa or Dapa on IL-6 release at 3µM or 10µM concentrations. Cells were pre-incubated for 16 h with vehicle or an SGLT2 inhibitor and subsequently stimulated by 1µg/mL LPS for 3 h with vehicle or an SGLT2 inhibitor at 3µM or 10µM concentration. IL-6 release into the cell media was determined for 10µM Empa (A, n=3), 10µM Dapa (B, n=3), 3µM Cana (C, n=4), 3µM Empa (D, n=4) and 3µM Dapa (E, n=4) treated HCAECs. Data are presented as mean±SD. *p<0.05, ***p<0.001, tested by one-way ANOVA with Bonferroni correction.

## Slide 3
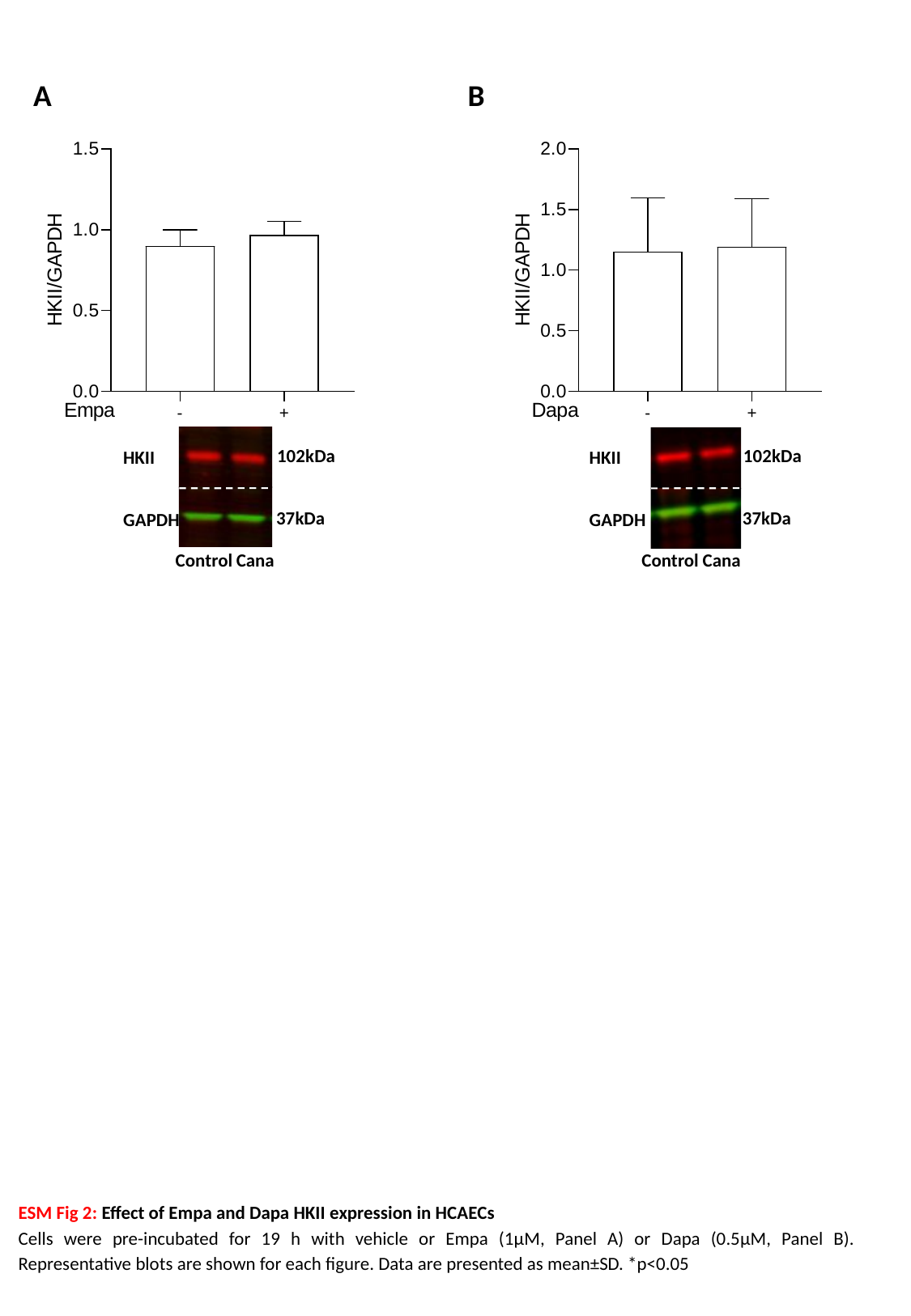

A
B
102kDa
HKII
37kDa
GAPDH
Cana
Control
102kDa
HKII
37kDa
GAPDH
Cana
Control
ESM Fig 2: Effect of Empa and Dapa HKII expression in HCAECs
Cells were pre-incubated for 19 h with vehicle or Empa (1µM, Panel A) or Dapa (0.5µM, Panel B). Representative blots are shown for each figure. Data are presented as mean±SD. *p<0.05

## Slide 4
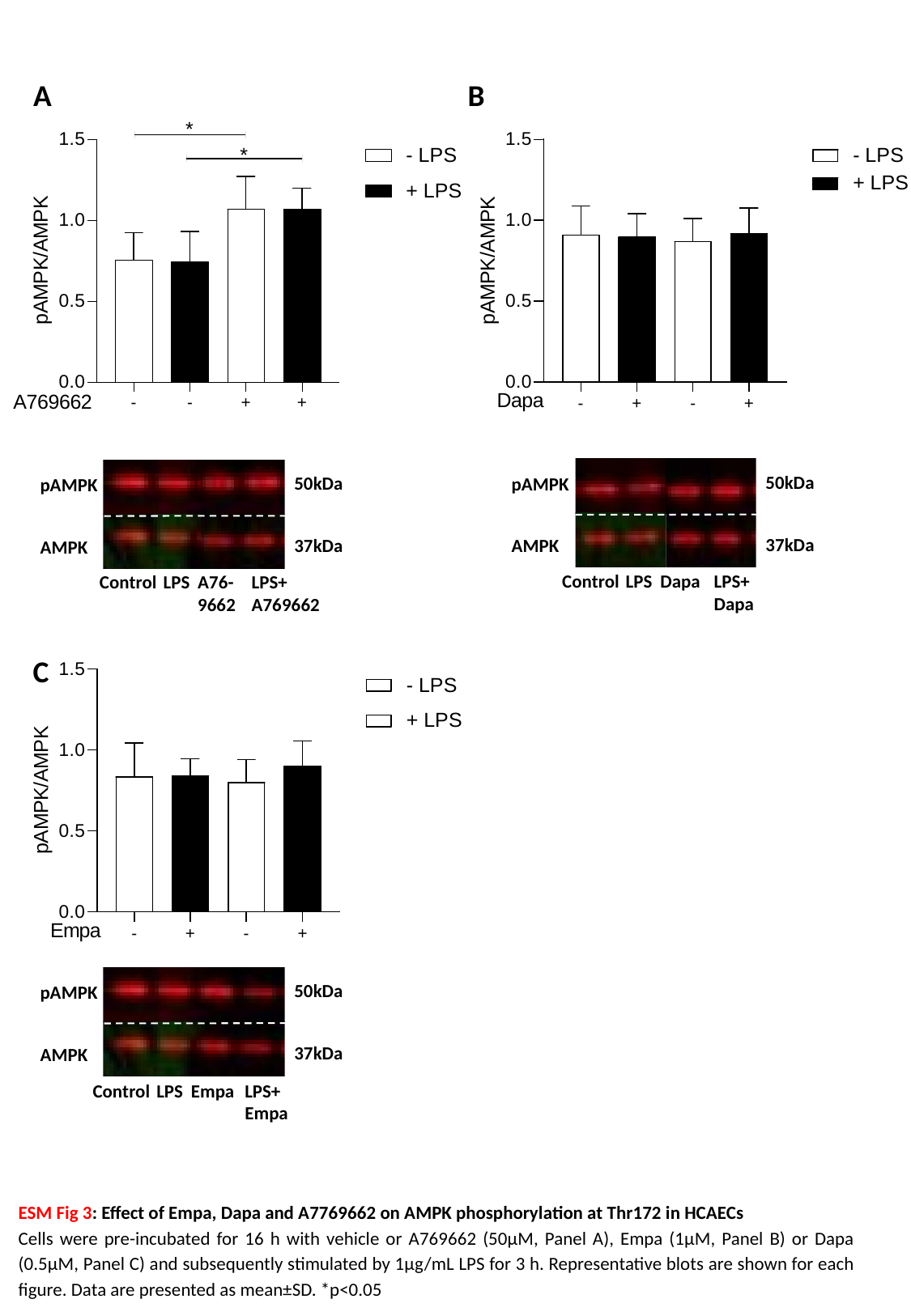

A
B
50kDa
pAMPK
37kDa
AMPK
Control
LPS
Dapa
LPS+
Dapa
50kDa
pAMPK
37kDa
AMPK
Control
LPS
A76-
9662
LPS+
A769662
C
50kDa
pAMPK
37kDa
AMPK
Control
LPS
Empa
LPS+
Empa
ESM Fig 3: Effect of Empa, Dapa and A7769662 on AMPK phosphorylation at Thr172 in HCAECs
Cells were pre-incubated for 16 h with vehicle or A769662 (50µM, Panel A), Empa (1µM, Panel B) or Dapa (0.5µM, Panel C) and subsequently stimulated by 1µg/mL LPS for 3 h. Representative blots are shown for each figure. Data are presented as mean±SD. *p<0.05

## Slide 5
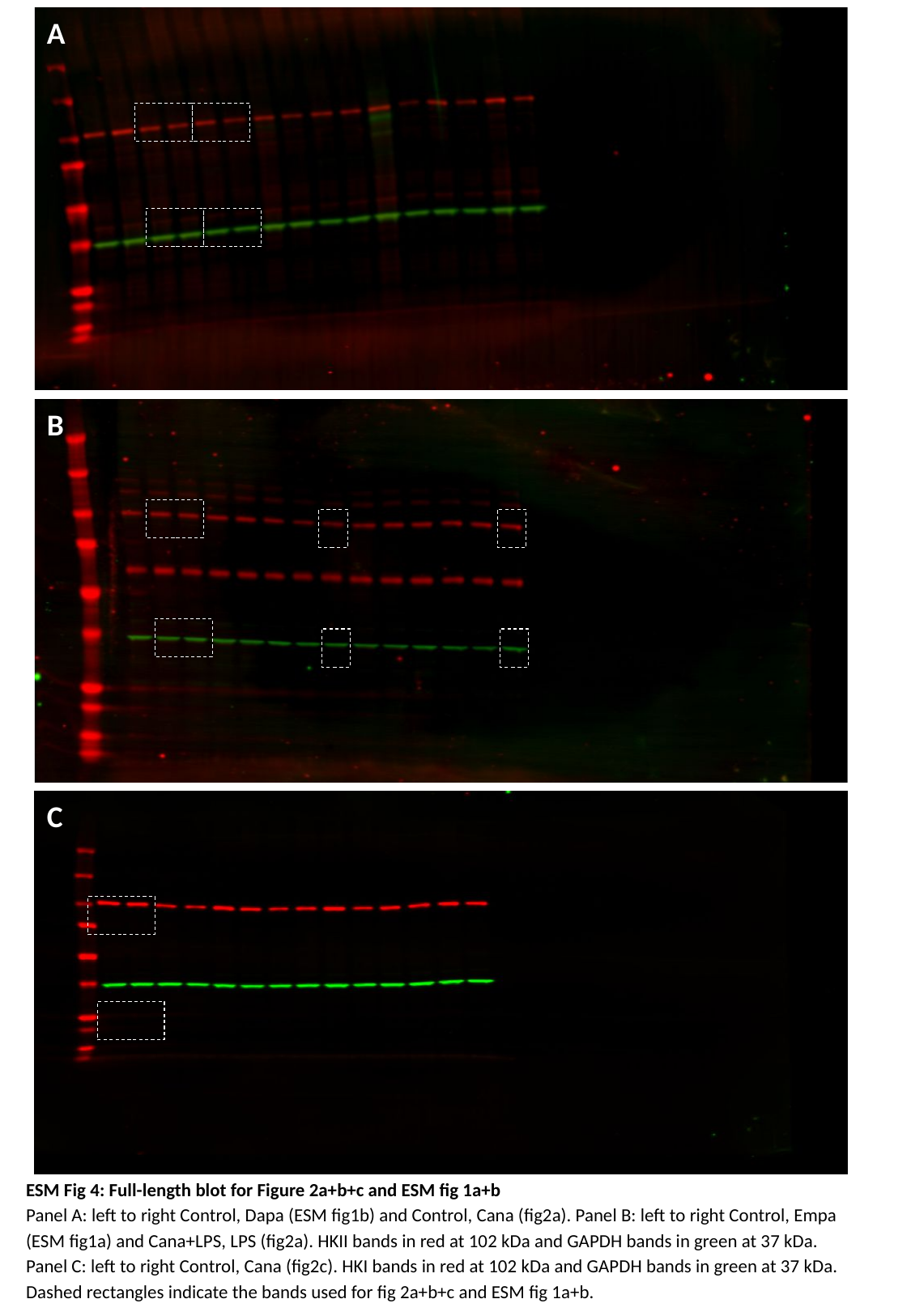

A
B
C
ESM Fig 4: Full-length blot for Figure 2a+b+c and ESM fig 1a+b
Panel A: left to right Control, Dapa (ESM fig1b) and Control, Cana (fig2a). Panel B: left to right Control, Empa (ESM fig1a) and Cana+LPS, LPS (fig2a). HKII bands in red at 102 kDa and GAPDH bands in green at 37 kDa.
Panel C: left to right Control, Cana (fig2c). HKI bands in red at 102 kDa and GAPDH bands in green at 37 kDa. Dashed rectangles indicate the bands used for fig 2a+b+c and ESM fig 1a+b.

## Slide 6
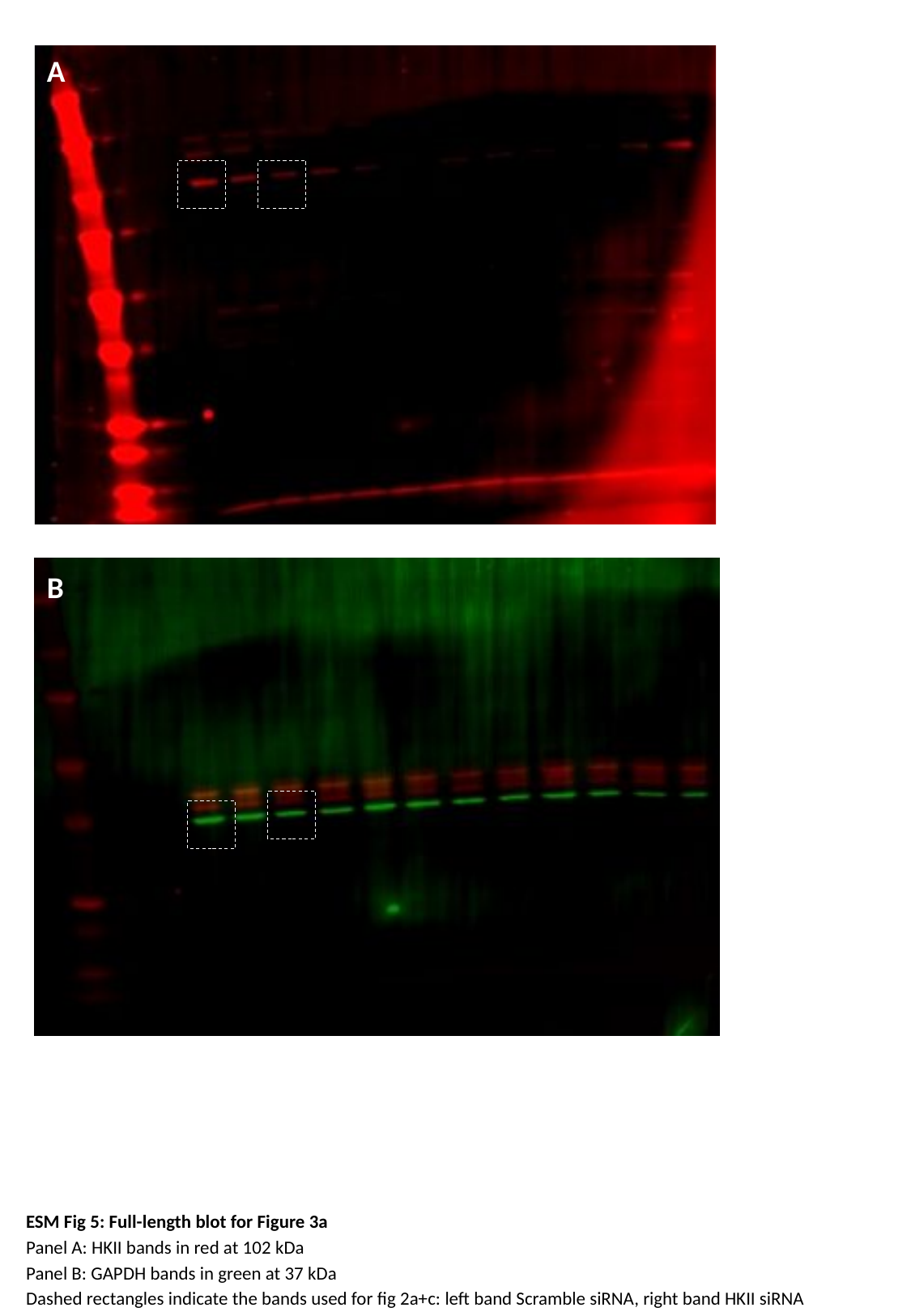

A
B
ESM Fig 5: Full-length blot for Figure 3a
Panel A: HKII bands in red at 102 kDa
Panel B: GAPDH bands in green at 37 kDa
Dashed rectangles indicate the bands used for fig 2a+c: left band Scramble siRNA, right band HKII siRNA

## Slide 7
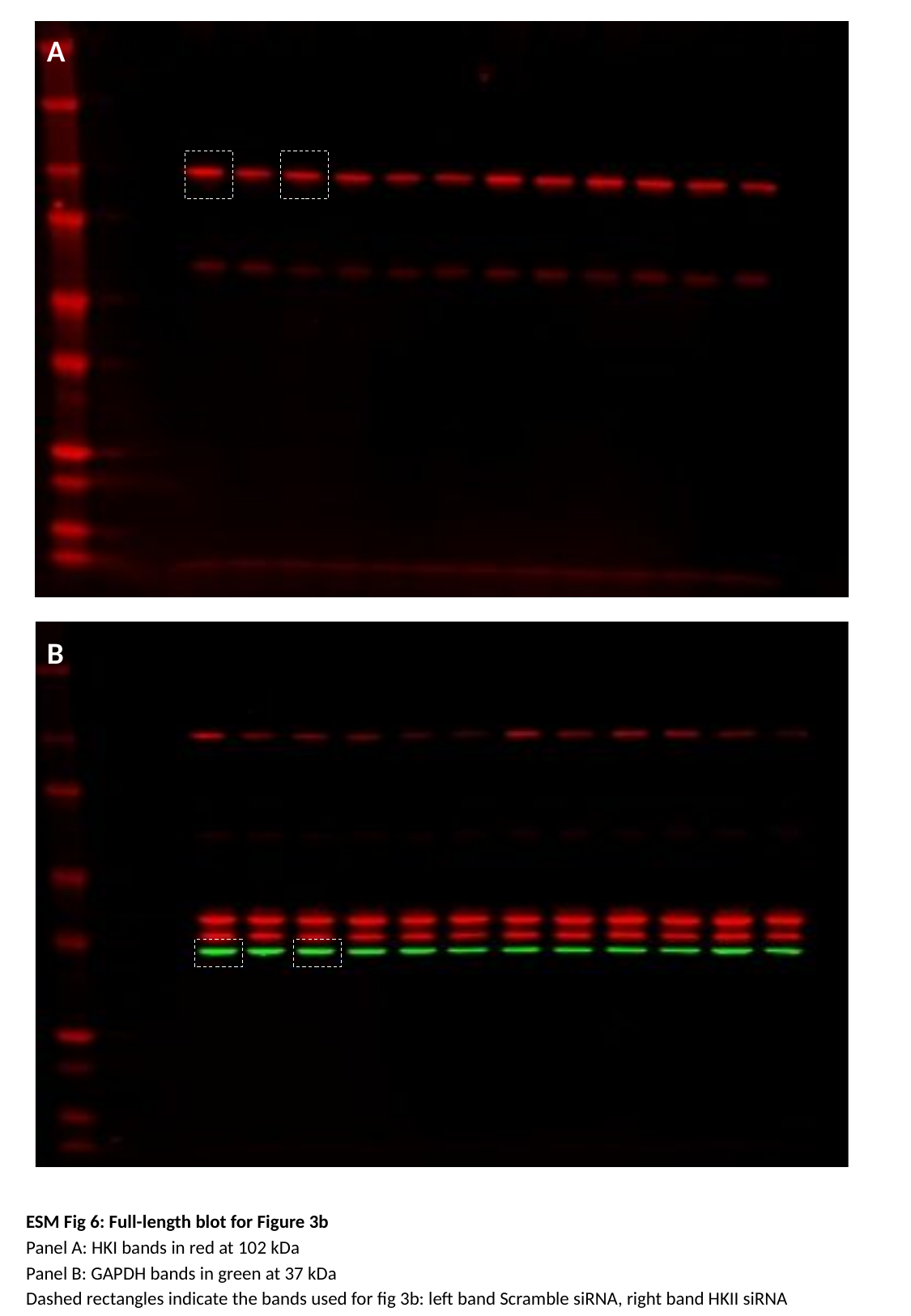

A
B
ESM Fig 6: Full-length blot for Figure 3b
Panel A: HKI bands in red at 102 kDa
Panel B: GAPDH bands in green at 37 kDa
Dashed rectangles indicate the bands used for fig 3b: left band Scramble siRNA, right band HKII siRNA

## Slide 8
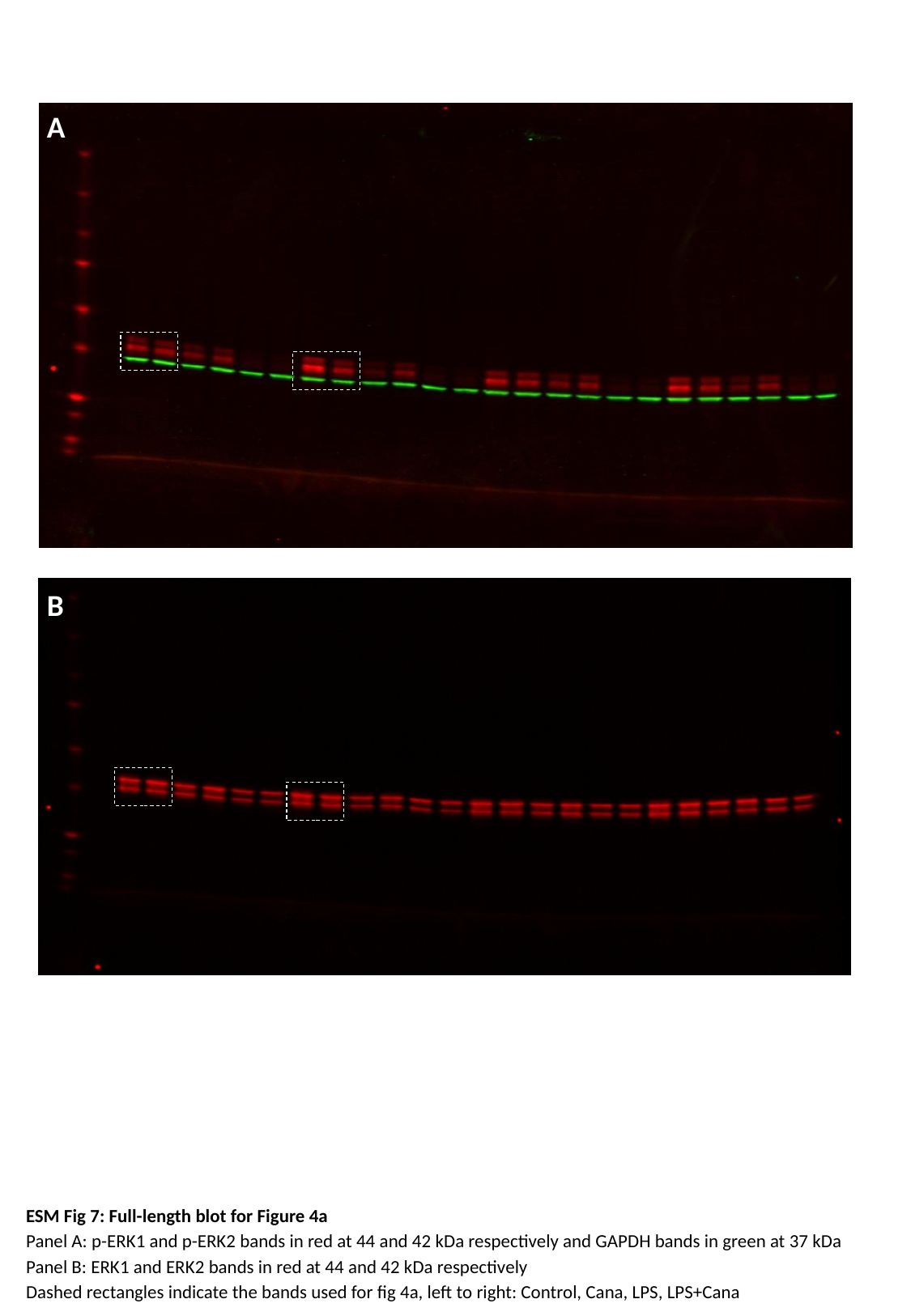

A
B
ESM Fig 7: Full-length blot for Figure 4a
Panel A: p-ERK1 and p-ERK2 bands in red at 44 and 42 kDa respectively and GAPDH bands in green at 37 kDa
Panel B: ERK1 and ERK2 bands in red at 44 and 42 kDa respectively
Dashed rectangles indicate the bands used for fig 4a, left to right: Control, Cana, LPS, LPS+Cana

## Slide 9
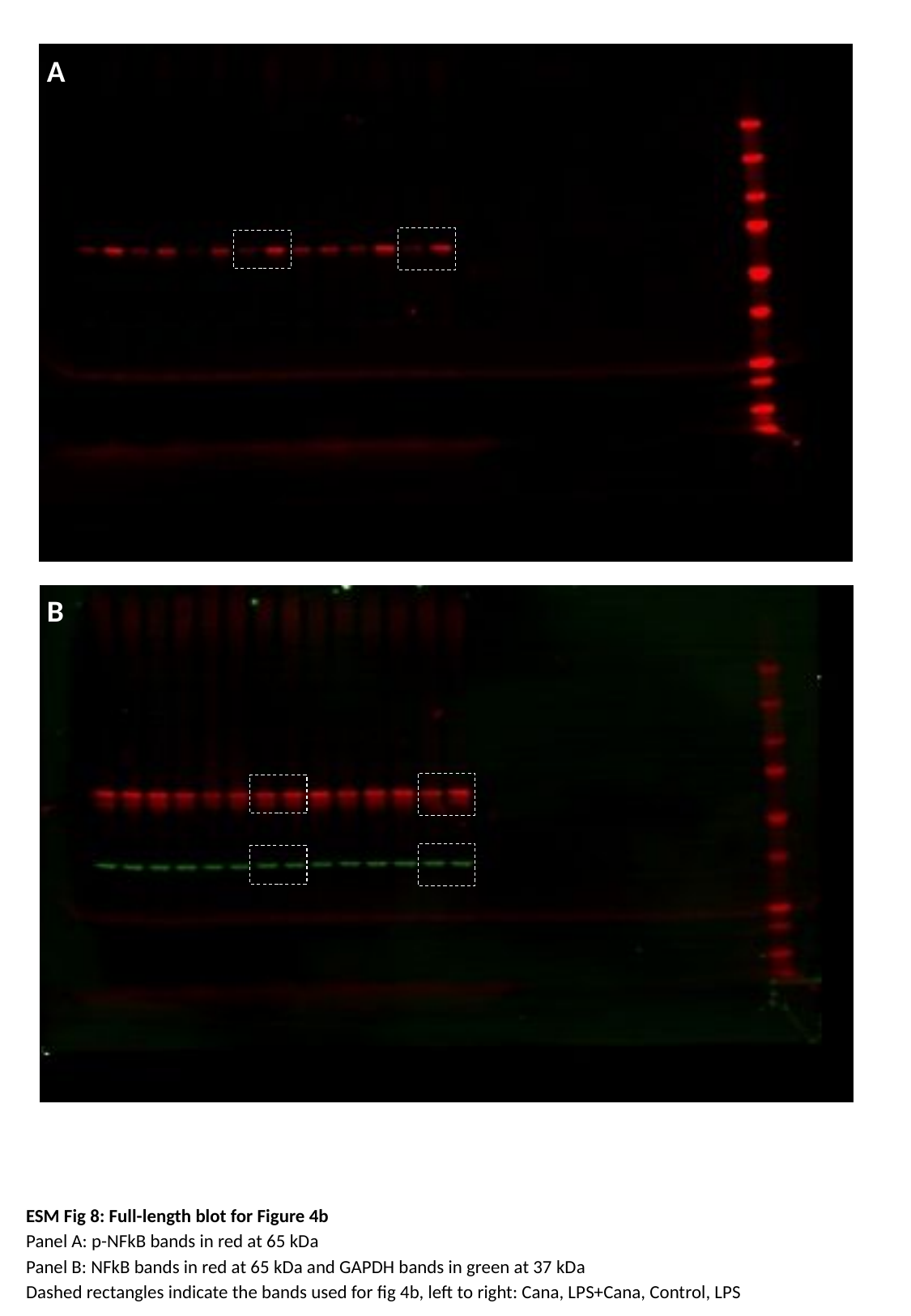

A
B
ESM Fig 8: Full-length blot for Figure 4b
Panel A: p-NFkB bands in red at 65 kDa
Panel B: NFkB bands in red at 65 kDa and GAPDH bands in green at 37 kDa
Dashed rectangles indicate the bands used for fig 4b, left to right: Cana, LPS+Cana, Control, LPS

## Slide 10
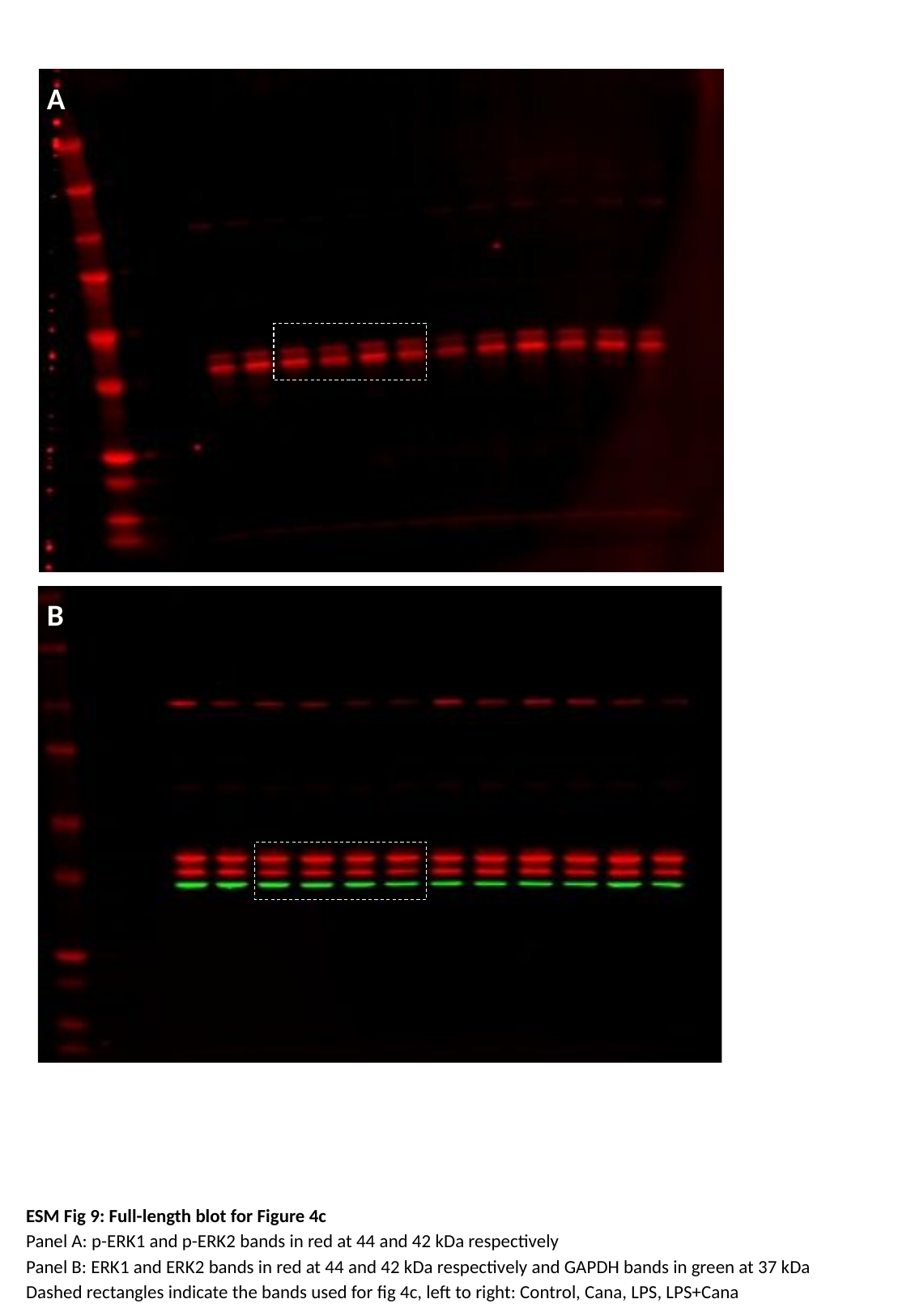

A
B
ESM Fig 9: Full-length blot for Figure 4c
Panel A: p-ERK1 and p-ERK2 bands in red at 44 and 42 kDa respectively
Panel B: ERK1 and ERK2 bands in red at 44 and 42 kDa respectively and GAPDH bands in green at 37 kDa
Dashed rectangles indicate the bands used for fig 4c, left to right: Control, Cana, LPS, LPS+Cana

## Slide 11
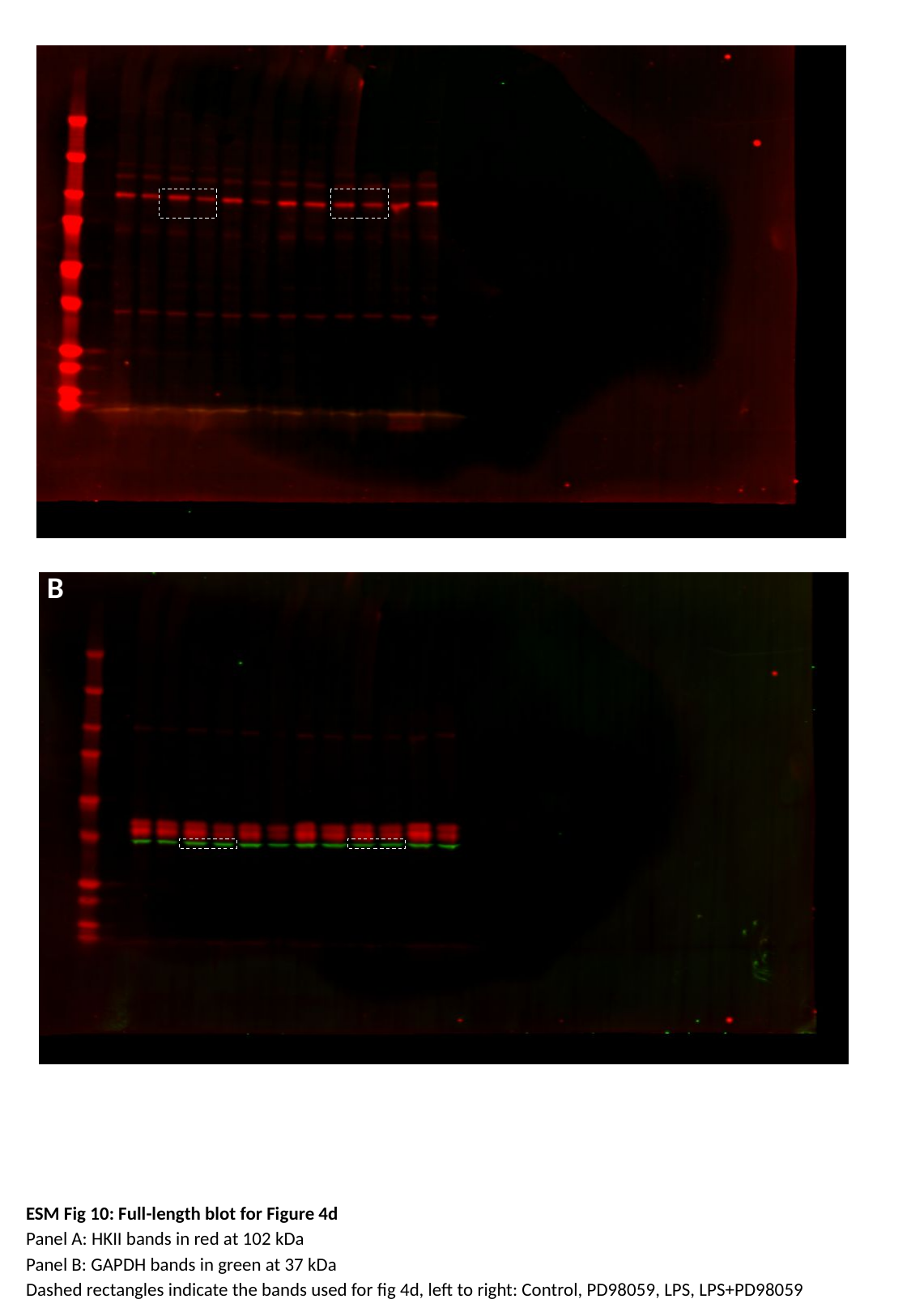

A
B
ESM Fig 10: Full-length blot for Figure 4d
Panel A: HKII bands in red at 102 kDa
Panel B: GAPDH bands in green at 37 kDa
Dashed rectangles indicate the bands used for fig 4d, left to right: Control, PD98059, LPS, LPS+PD98059

## Slide 12
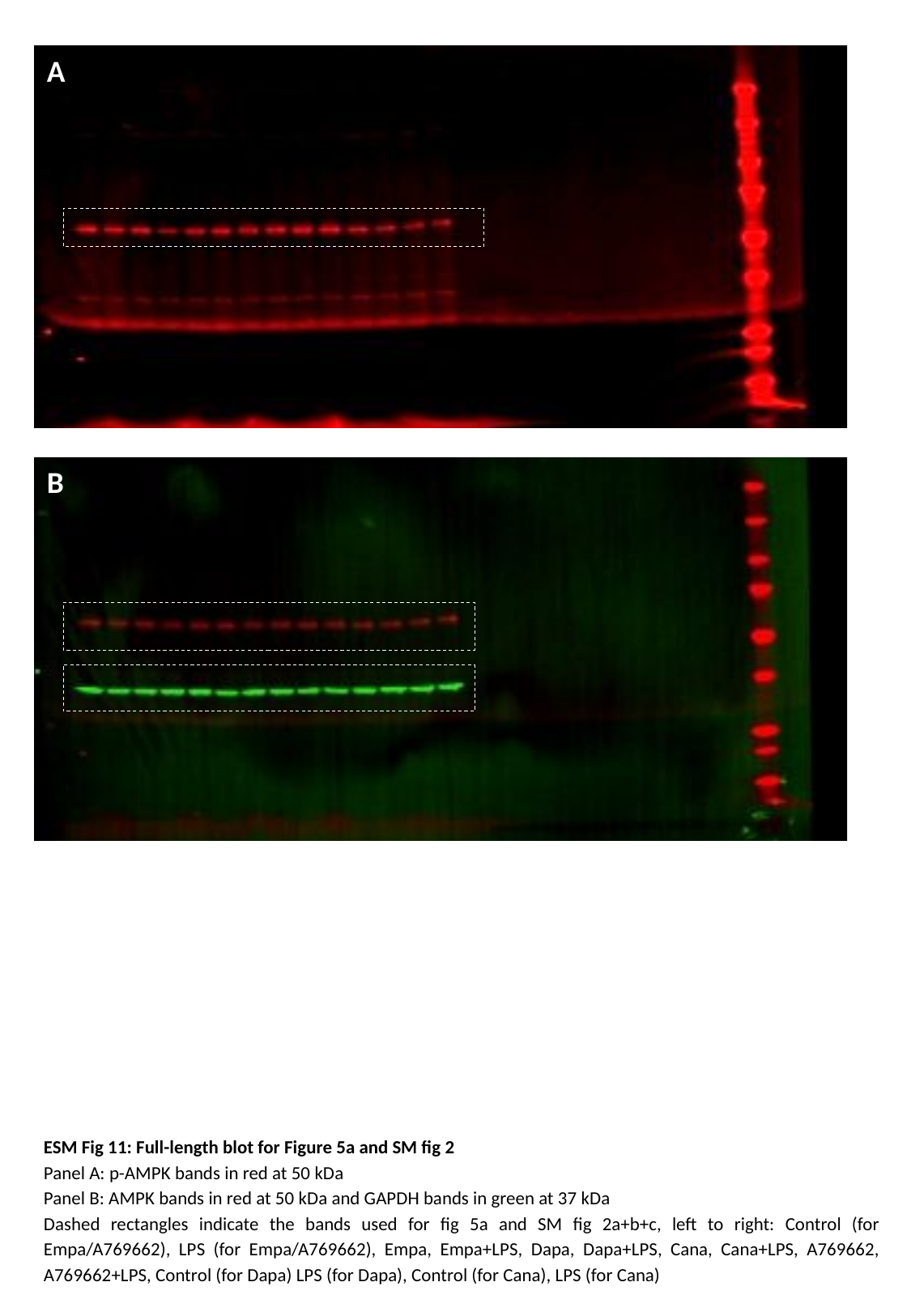

A
B
ESM Fig 11: Full-length blot for Figure 5a and SM fig 2
Panel A: p-AMPK bands in red at 50 kDa
Panel B: AMPK bands in red at 50 kDa and GAPDH bands in green at 37 kDa
Dashed rectangles indicate the bands used for fig 5a and SM fig 2a+b+c, left to right: Control (for Empa/A769662), LPS (for Empa/A769662), Empa, Empa+LPS, Dapa, Dapa+LPS, Cana, Cana+LPS, A769662, A769662+LPS, Control (for Dapa) LPS (for Dapa), Control (for Cana), LPS (for Cana)

## Slide 13
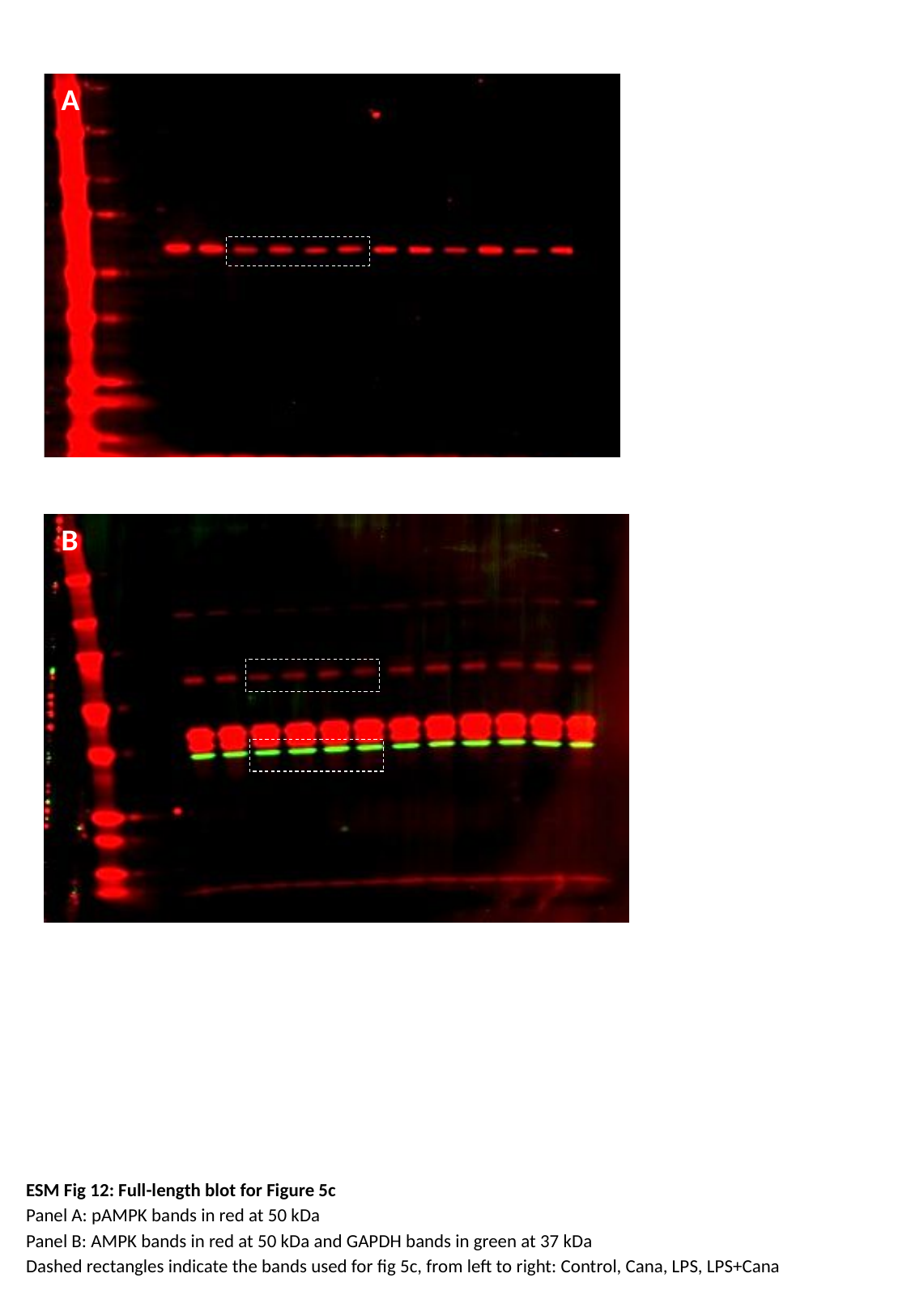

A
B
ESM Fig 12: Full-length blot for Figure 5c
Panel A: pAMPK bands in red at 50 kDa
Panel B: AMPK bands in red at 50 kDa and GAPDH bands in green at 37 kDa
Dashed rectangles indicate the bands used for fig 5c, from left to right: Control, Cana, LPS, LPS+Cana
